# Magnitude and predictors of female domestic abuse in pregnancy in a patriarchal African society: a cross-sectional study of pregnant women in Enugu, South East Nigeria

**DOI:** 10.11604/pamj.2021.40.82.28157

**Published:** 2021-10-07

**Authors:** Ifeoma Veralyn Njoku, Joseph Tochukwu Enebe, Cyril Chukwudi Dim

**Affiliations:** 1 Department of Obstetrics and Gynaecology, University of Nigeria Teaching Hospital, Ituku-Ozalla, Enugu, Nigeria,; 2 Department of Obstetrics and Gynaecology, Enugu State University of Science and Technology College of Medicine, Teaching Hospital, Parklane, Enugu, Nigeria,; 3 Department of Obstetrics and Gynaecology, College of Medicine, University of Nigeria, Ituku-Ozalla Campus, Enugu, Nigeria

**Keywords:** Pattern, predictors, domestic abuse, pregnant women, Enugu, Nigeria

## Abstract

**Introduction:**

domestic abuse against women is very common globally and has far-reaching consequences on the society. Therefore, it is essential to deeply study the seriousness of this public health issue among our pregnant women. The objectives were to determine the prevalence, pattern, and predictors of domestic abuse among pregnant women in Enugu, Nigeria.

**Methods:**

a cross-sectional study of 400 consenting pregnant women at the antenatal clinics of the University of Nigeria Teaching Hospital Ituku-Ozalla, Nigeria. Each woman completed a modified abuse assessment screen structured questionnaire. Data analysis was descriptive and inferential with Chi-square and multivariate binary logistic regression using SPSS version 21. A p-value of <0.05 was considered statistically significant.

**Results:**

a total of 172 out of 400 respondents (43.0%) had ever experienced domestic abuse in their pregnancies. One hundred and ten (37.2%; 110/296) of the multigravid women were abused in previous pregnancies while 137 (34.3%; 137/400) of all respondents were being abused in the current pregnancy. For all pregnancies, the most common type of abuse experienced by the respondents was verbal abuse (85.5%; 147/172), while the commonest perpetrators of abuse were the respondents´ spouses for both the index pregnancy (82.5%; 113/137) and previous pregnancies (84.5%; 93/110). The most common women perceived cause of abuse was financial constraints (68.6%; 118/172). Age less than 25 years (AOR=1.9, 95% CI=1.01-3.76, p=0.048), not having tertiary education (AOR=2.0, 95% CI=1.17 - 3.25, p=0.01), having at least a male child (AOR=3.3, 95% CI=1.71 - 6.40, p=<0.001), and maternal unemployed status (AOR=2.0, 95% CI=1.27 - 3.19, p=0.003) were the identified predictors among women abused in pregnancy.

**Conclusion:**

the prevalence of domestic abuse was high among pregnant women in the University of Nigeria Teaching Hospital Ituku-Ozalla, Enugu, Nigeria. The predominant women perceived cause of domestic abuse was financial constraints while age less than 25 years, not having tertiary education, having at least a male child, and maternal unemployed status were the predictors in abused women. Domestic abuse poses a great threat to women´s reproductive health, and so, its screening should be incorporated into antenatal care in our environment.

## Introduction

Domestic abuse otherwise referred to as domestic violence, is defined by the United Nations as any act of gender-based violence that results in physical, sexual or psychological harm or suffering, including threats of acts such as coercion or arbitrary deprivation of liberty, whether occurring in public or private life [1]. The most common victims of domestic abuse are women, [2,3] and it has been shown that about 5% of healthy years of life lost by women in developing countries are due to domestic abuse [4]. Domestic abuse against women and children is regarded as a public health crisis, and it has serious effects on the psycho-social life of individuals and families globally [5,6]. Also, during pregnancy, domestic abuse puts the lives of both the mother and her unborn child at risk.

Worldwide, the prevalence of domestic abuse varies significantly, but it is worse in developing nations, reflecting the genuine diversity in the definition of abuse in pregnancy by both the victims and the researchers [7]. Globally, at least 35% of women have experienced some form of domestic abuse [5,8]. In pregnancy, domestic abuse has been more prevalent in low-income (27.7%) countries than high-income ones (13.3%) [9]; for instance, the prevalence was 6-9% in New Zealand [10], 31.4% in Japan [11] and 48.2% in India [12]. Unfortunately, Africa seems to have the highest reported domestic abuse in pregnancy rates [13]; a report from Ethiopia showed a very high rate of 78.5% among pregnant women [14]. In Nigeria, the prevalence of domestic abuse in pregnancy ranged from 5 to 58.6% [3,15-18], and this wide range might be due to the lack of a standard method for its estimation.

Furthermore, different forms of domestic abuse in pregnancy were reported in various centres in Nigeria. Physical abuse was the most common form of domestic abuse in pregnancy, and it was found in 58.6% in Kano [15], 58.9% in Imo [19], and 50.9% in Jigawa [20]. Other dominant forms of abuse in other centres were: forced sexual intercourse found in 65% of pregnant women in Jos [21], verbal/psychological abuse in Abuja (66.4%) [17], and Jigawa (68.4%) [20]. In Enugu, Ezegwui *et al*. [18] reported that 13.1% of the pregnant women interviewed had physical beating during their pregnancies while 60% had verbal abuse as the major abuse from their spouses. Also, intimidation, threats of bodily harm, acid baths, withdrawal of financial support, social isolation, etc. were reported by pregnant women in some studies [20,22,23]. In particular, the isolation of the victim from her family or friends prevented the latter from detecting the abuse, thus increasing the woman´s total dependence on her abuser and increasing the risk of other forms of abuse developing [22].

Domestic abuse of a pregnant woman, especially physical violence, increases the risk of complications to the mother and her unborn child. It could lead to preterm labour [21], eating disorders, low maternal weight gain, depression, alcoholism, drug abuse, anaemia, infections, a late start for antenatal care, miscarriages, preterm deliveries, low birth weight, perinatal and even maternal death [22-24]. Intimate partners, usually the husbands, are responsible for about 38% of homicides involving women globally, and most of them occur during pregnancy [5]. Jealous co-wives or disapproving in-laws were also culprits, and sometimes they do this out of their own volition or in league with the victim´s spouse [20,21].

Often, domestic abuses in pregnancy situations go unreported because the victims decide to keep them a secret due to socio-cultural influences or the fear of the repercussions from their abuser(s) should they report such incidences [21]. Many other factors have been blamed as reasons for the occurrence of domestic abuse in pregnancy in some homes. These factors include differences in ethnicity of a couple, differences in religion, low educational status of either spouse, no/low employment status, polygamy, disease (human immunodeficiency virus (HIV), poverty, unsanitary living conditions, lack of medical care, no male offspring, alcoholism, drug abuse, unplanned pregnancy, rejection of a partner´s sexual advances, financial constraints, etc. [15,18,20,21]. In Enugu, Nigeria, financial constraints were the most common cause of domestic abuse in pregnancy [18].

While domestic abuse against pregnant women is seen globally as a violation of human rights, there are unfortunately provisions in the Nigerian constitution that make it legal to abuse women [25,26]. For instance, the wife´s beating by her husband to correct her is permitted according to section 55 (1d) of the penal code [21,24], while section 182 does not recognize marital rape [24]. Although Nigeria is a signatory to many international women´s rights charter agreements such as the elimination of discrimination against women, none can go into effect nationally unless parliament signs a corresponding anti-domestic abuse law or repeals the old penal laws [24]. Fortunately, in 2015, the gender-based “Violence against Persons (Prohibition) Act (VAPP Act) bill was finally signed into law by the senate after more than ten years of undergoing legislative review” [25]. In it, several sections cover the prohibition against and punishment for different types of domestic abuse against women [25]. The law also allows an order of protection for women who are victims if they complain or report their abuse and gives harsher punishments for sexual violence. But the law is yet to be fully implemented at both the federal and state levels mostly because the populace and the law enforcement agencies are not fully aware of this relatively new and under-publicized law, and the penal laws of the 1999 constitution are yet to be repealed [25]. This study assessed whether or not the respondents were aware of this law or any other laws in Nigeria that prohibit domestic abuse against women. The study also gauged whether the respondents were aware of any non-governmental organizations that provide support and legal aid for victims of domestic abuse such as the Women´s Rights Advancement and Protection Alternative (WRAPA) and Women´s Aid Collective (WACOL) [25].

From recently published reports from other centres in Nigeria, domestic abuse is still prevalent in our society and may even be rising [16,20,27]. The last published report from the study area was more than a decade ago [18]. Based on their findings, the authors had suggested incorporating routine domestic abuse screening at the antenatal clinic to highlight the problem and set up protocols that would provide aid to affected women. That recommendation is yet to be carried out. Thus, there was a need to re-assess this maternal health problem to determine its current prevalence, pattern, predictors, and perceived complications in pregnancy. Other study objectives were to determine women´s views about introducing a routine screening of domestic abuse during antenatal care visits, perception of how to manage domestic abuse, and their opinion on routine domestic abuse screening at the antenatal clinics.

## Methods

### Study design: this was a cross-sectional study

**Study setting:** this study was carried out at the antenatal clinic of the University of Nigeria Teaching Hospital (UNTH) Ituku-Ozalla, Enugu, Nigeria, between January and March 2017. The hospital was randomly selected among the two teaching hospitals offering maternity services in Enugu Metropolis, Nigeria. The consultant-led antenatal clinic operates every weekday from Monday to Friday. On average, 638 pregnant women attend the clinic every month, of which about 128 were first visits, and 510 were re-visits.

**Study participants:** all consenting pregnant women, irrespective of gestational age, that attended the antenatal clinics of the study centre within the study period were eligible for the study. All pregnant women who did not give their consent for this study and those that were so ill that their mental capacity could not allow them to easily understand the questions in the questionnaire were excluded from this study.

**Outcome measures:** the primary outcome of the study was the prevalence of domestic abuse in pregnancy among the respondents.

**Sampling techniques:** systematic sampling was used to select the sample population. A sampling interval of five was adequate to achieve the study´s sample size within the study period. Thus, at each antenatal clinic day, a frame of all registered attendees for that day was obtained, and a random start was selected by simple random sampling. Then, starting with the woman corresponding to the random start, every subsequent fifth name on the sampling frame was systematically selected. Each selected woman was identified and counselled for the study.

**Study instrument:** the study instrument was a pre-tested structured questionnaire adapted from the Abuse Assessment Screen Tool (AASC) developed by McFarlane and Parker [28]. The AASC is the oldest domestic violence screening tool still used today in the United States [29]. It is used as an aid for the detection of abuse of women, mainly during pregnancy. It has a sensitivity of 93-94%, and a specificity of 65-99% and a high test-retest reliability and predictive validity based on accepted diagnostic criteria set forth by the United States Preventive Services Taskforce (USPST) [26]. McFarlane´s original questionnaire consisted of only five questions. The questionnaire used for this study was in two sections. Section A contained questions designed to gather sociodemographic information about the study population. Section B had modified questions designed to elicit information about the type(s) of domestic abuse or lack thereof experienced by the participants, the perpetrators, and the risk factors/predictors. Each question asked in section B was a modification of the original questions posed/written by McFarlane and with added sub-questions covering experiences of physical, sexual, and emotional violence. The questionnaire was first pre-tested among female medical doctors and a cohort of randomly selected pregnant women to ensure clarity of language, appropriateness, and sufficiency and remove bias. Trained assistants comprising of five medical doctors distributed the questionnaire to consenting women. The respondents were assured of the anonymity and confidentiality of their responses to encourage them to answer honestly.

The prevalence of domestic abuse in pregnancy was obtained in this study based on the definition by the United Nations as any act of gender-based violence that results in physical, sexual or psychological harm or suffering, including threats of acts such as coercion or arbitrary deprivation of liberty, whether occurring in public or private life [1].

**Data collection:** pregnant women were addressed as a group at the antenatal clinics by the researchers on the rationale of the study and the likely benefits and harms. During this introduction, the participants were given the opportunity to ask questions and clarifications were given. Written informed consent was obtained from each respondent by the investigators before the administration of the questionnaire. The contents, frame and expectations of the questionnaire were further carefully explained to each of the participants without answering the questions in the questionnaires. The researchers offered assistance to respondents that needed more clarification while filling the questionnaires. The questionnaires were self-administered; however, the interviewer-administered option was used for a few respondents that still had difficulties understanding the questions despite previous explanations.

**Sample size determination:** a total of 422 pregnant women was the minimum sample size calculated for this study. This was determined using the Cochrane formula for minimum sample size determination in a cross-sectional study [30];


$$n=Z^{2}pq/d^{2}$$

The calculation assumed a domestic abuse in pregnancy prevalence of 50% (p=0.5, q=1-p), standard normal deviate (Z) corresponding to a 95% level of significance or confidence interval of 1.96, the precision (d) of 5%, and a non-response rate of 10%.

**Statistical analysis:** the data collected was analyzed using the Statistical Package for Social Sciences (SPSS) version 21. Complete case analysis was done. Univariate statistics were used to analyze quantitative and qualitative variables. Means and standard deviations were reported for numeric variables while frequencies, percentages and proportions were used to report categorical variables. Inferential statistics were carried out at a 95% confidence level. A p-value of < 0.05 was considered statistically significant. Proportions were compared using the Chi-square or Fisher´s exact test, where applicable. Further analysis was done using multivariate binary logistic regression (all factors with a Chi-square p-value < 0.25 were used to build the multivariate regression analysis) to determine the predictors of respondent reported experience of domestic abuse in pregnancy.

**Informed consent:** written informed consent was obtained from every participant before they were enrolled on the study.

**Ethical approval:** approval for this study with certificate number NHREC/05/01/2008B-FWA00002458-1RB00002323 was obtained from the Clinical and Ethics Committee of UNTH.

## Results

**Socio-demographic characteristics of the study participants:** a total of 422 pregnant women were recruited for the study, but 400 of them completed the questionnaires correctly and returned them, which gave a response rate of 94.8%. The mean age of the respondents was 30.1 ± 2.47 (range: 20-45) years. A majority of the respondents were Igbos (92.5%), 30-34 years (42.5%) of age, Christians (96.5%), multigravida (74.0%), and had tertiary education (67.0%). Three hundred and ninety (96.5%) respondents were married, and out of that number, 376 (94.0%) women were in monogamous marriages. The respondents' mean gestational age was 25.4 ± 8.12 (range 20 - 40) weeks. The details of the socio-demographic characteristics of the respondents are shown in Table 1.

**Table 1:** basic characteristics of respondents

Characteristics (n=400)	Sub-groups	Frequency	Percent
Age group (years)	≤19	0	0.0
	20 - 24	60	15.0
	25 - 29	141	35.3
	30 - 34	170	42.5
	35 - 39	29	7.2
	≥ 40	0	0.00
Religion	Christianity	386	96.5
	Islam	14	3.5
Ethnicity	Igbo	370	92.5
	Yoruba	20	5.0
	Hausa/Fulani	8	2.0
	Others	2	0.5
Educational level	Primary	17	4.3
	Secondary	106	26.5
	Tertiary	268	67.0
	No formal education	9	2.3
Marital status	Single	10	2.5
	Married	386	96.5
	Separated	4	1.0
Marital setting	Monogamous	376	94.0
	Polygamous	14	3.5
	Not applicable	10	2.5
Duration of pregnancy (weeks)	1 - 13	86	21.5
	14 - 27	99	24.8
	28 - 40	208	52.0
	Unsure	7	1.7
Gravidity	Multigravida	296	74.0
	Primigravida	104	26.0
No. of male children (n=296)*	None	64	21.6
	1	99	33.4
	2	77	26.0
	3	52	17.6
	4or more	4	1.4
Job status of respondent	Employed	233	58.3
	Unemployed	167	41.8
Job status of spouse/partner	Employed	371	92.8
	Unemployed	29	7.3

* Primigravida excluded

**Prevalence of domestic abuse among respondents:** of the 400 respondents, 172 women reported having ever been abused in pregnancy, which gave an overall domestic abuse prevalence of 43.0% (95%CI: 38.2 - 47.9). Of the 172 women who reported abuse in this study, 137 (34.3%; 137/400) were being abused in the index pregnancy. Out of the 296 multigravid respondents, 110 (37.2% (95%CI: 31.9 - 42.8)) had experienced abuse in previous pregnancies. The most common perpetrators of the abuse to respondents were their spouses for both the index pregnancy (82.5%; 113/137) and previous pregnancies (84.5%; 93/110). Other perpetrators of domestic abuse in the index pregnancy were the mother-in-law (18.2%; 27/137), sister-in-law (8.0%; 11/137), and neighbour (2.9%; 4/137). For the previous pregnancies, the other perpetrators of domestic abuse were the mother-in-law (10.0%, 11/110), sister-in-law (6.4%, 7/110) and a co-wife (0.9%, 1/110).

**The pattern of domestic abuse among respondents:** Table 2 shows the types of domestic abuse suffered by respondents. For all pregnancies, the most common type of abuse was verbal abuse (85.5%, 147/172), while the least common was forced sexual acts (7.3%, 10/172); a similar pattern of abuse was observed for the index pregnancy.

**Table 2:** types of abuse in pregnancy (multiple responses allowed)

Type of abuse	All pregnancies (n=172)		Index pregnancy (n=137)	
	Frequency	Percent	Frequency	Percent
Physical battery	77	44.8	44	32.1
Verbal insults	147	85.5	102	74.5
Forced sexual acts	10	7.3	11	8.0
Threats of bodily harm	59	34.3	41	29.9
Withdrawal of financial support	83	48.3	52	37.9

**Perceived complications suffered in pregnancy secondary to abuse:** concerning the perceived complications of domestic abuse in pregnancy, 76 (69.1%) respondents abused in previous pregnancies believed they had suffered complications related to their abuse, and these included preterm labour (48.7%, 37/76), preterm delivery (5.3%, 4/76), bleeding per vagina (1.3%, 1/76), miscarriage (52.6%, 40/76) and depression (90.8%, 69/76).

**Predictors of domestic abuse among respondents:** Table 3 shows the respondents perceived causes of their abuse in pregnancy - responses here were multiple for many respondents (n = 172). A majority (68.6%; 118/172) of respondents felt that financial constraints were the most predominant cause of abuse, followed by having no male child (61.1%; 105/172). A different ethnicity between the couple was considered the least reason for abuse (3.5%; 6/172). Table 4 shows the details of the association between respondents´ basic characteristics and the prevalence of domestic abuse in pregnancy. Multivariate logistic regression for possible predictors of domestic abuse among respondents showed that age less than 25 years, not having a tertiary education, having at least a male child and unemployed status of respondents predicted domestic abuse during pregnancy. Domestic abuse during pregnancy was about 2 times higher among respondents whose ages were <25 years than those that were 25 years or more (OR = 1.9 [95%CI 1.01 - 3.76]); and 3 times higher among women who had at least a male child than those without a male child (OR = 3.3 [95%CI 1.71 - 6.40]). Also, domestic abuse in pregnancy was 2 times higher among women with less than tertiary education (OR = 2.0 [95%CI 1.17 - 3.25]) and those without any employment (OR = 2.0 [95%CI 1.27 - 3.19]) compared to those with tertiary education and those that were employed, respectively.

**Table 3:** respondents’ perceived causes of their abuse in pregnancy (multiple responses allowed) (n=172)

Perception of cause of abuse	No. of respondents	Percent
Earn more than spouse	8	4.7
Financial constraints	118	68.6
Spouse unemployed	40	23.3
Unplanned pregnancy	25	14.5
No male child	105	61.1
Higher educational level than spouse	9	5.2
Different ethnicity	6	3.5
Drug abuse/alcoholism	36	20.9
HIV-positive status	10	5.8
Disapproving in-laws	30	17.4

**Table 4:** relationship between the prevalence of domestic abuse and maternal characteristics

Variable	Sub-group	Ever abused		##P-value	OR (95%CI)	**aOR (CI95%)	P-value
		**Yes**	**No**				
		**Freq (%)**	**Freq (%)**				
Age groups (years)	<25	34 (56.7)	26 (43.3)	0.020	1.9 (1.10-3.33)	1.9 (1.01-3.76)	0.048
	>=25	138 (40.6)	202 (59.4)				
Religion	Islam	10 (71.4)	4 (28.6)	*0.051	3.5 (1.07-11.22)	0.6 (0.083-4.89)	0.663
	Christianity	162 (42.0)	224 (58.0)				
Ethnicity	Non-Igbo	20 (66.7%)	10 (33.3)	0.007*	2.9 (1.31-6.30)	2.4 (0.82 - 7.07)	0.107
	Igbo	152 (41.1)	218 (58.9)				
Education status	Less than tertiary	72 (54.5)	60 (45.5)	0.001	2.0 (1.32-3.08)	2.0 (1.17 - 3.25)	0.01
	Tertiary	100 (37.3)	168 (62.7)				
Marital setting	Polygamous	9 (64.3%)	5 (35.7)	0.168*	2.4 (0.80-7.39)	1.8 (0.47 - 6.80)	0.398
	Monogamous	160 (42.6)	216 (57.4)				
Gravidity	Multigravida	145 (49.0)	151 (51.0)	<0.001	2.7 (1.67-4.49)	1.31 (0.62 - 2.76)	0.488
	Primigravida	27 (26.0)	77 (74.0)				
Have male child	At least a male child	129 (55.4)	104 (44.6)	<0.001	3.6 (2.32-5.51)	3.3 (1.71 - 6.40)	<0.001
	No male child	43 (25.7)	124 (74.3)				
Women's job status	Unemployed	84 (50.3)	83 (49.7)	0.013	1.7 (1.12-2.49)	2.0 (1.27 - 3.19)	0.003
	Employed	88 (37.8)	145 (62.2)				
Spouse job status	Unemployed	9 (31.0)	20 (69.0)	0.242*	0.6 (0.26-1.30)	0.5 (0.21 - 1.38)	0.195
	Employed	163 (43.9)	208 (56.1)				

##P-value=p value of bivariate analysis; *Fisher exact test; ** aOR from multinomial logistic regression; # primigravida excluded

**Awareness of existing laws against domestic abuse:** only 85 (21.3%, 85/400) of respondents were aware that there were laws in Nigeria that prohibited domestic abuse. Out of all the listed non-governmental organizations that offer help and support to abused women, 30.3% (121/400) of all the respondents were aware of the Women´s Aid Collective (WACOL). Other organizations recognized by respondents were the Project Alert on Violence Against Women (13.8%, 55/400), the Women´s Rights Advancement and Protection Alternative (WRAPA) (16.3%, 65/400), and the Domestic Violence and Abuse Resource Centre Abuja (7.3%, 29/400).

**Women´s perception of how to manage domestic abuse:** some of the respondents (23, 5.8%) believed the victim should endure/do nothing, physically fight back (38, 9.5%) and verbally abuse the perpetrator (6, 1.5%). Only 18.3% (73/400) believed that abuse could be reported to the police. In comparison, 30.3% (121/400) of the women thought that reporting the abuse to family elders was the best way to handle the situation. Only 8 (4.7%) of the 172 women ever abused in pregnancy had reported the matter to the police. However, in 4 (50.0%) of the reported cases, the perpetrator was arrested but later released without prosecution.

**Respondents´ opinion on incorporating domestic abuse screening at the antenatal clinics:** sixty-one percent (244/400) of the respondents believed that pregnant women should be screened for domestic abuse at the antenatal clinics, 7.8% (31/400) disagreed with routine screening as they felt it was a private family matter; the remaining 31.3% (125/400) were unsure of its benefits.

## Discussion

This study found an overall domestic abuse in pregnancy prevalence of 43.0% while the most common type of abuse in pregnancy was verbal abuse (85.5%). Furthermore, as much as 69.1% of women abused in previous pregnancies believed they suffered complications related to their abuse. Also, the predictors of domestic abuse in pregnancy identified by the study were women´s age less than 25 years, not having a tertiary education, having at least a male child, and unemployed status. The finding that about 1 in 3 women attending antenatal care at the study centre during the study period had experienced domestic abuse in pregnancy in one form or another, suggests that such abuse is relatively common in the study area. The domestic abuse prevalence of 43% identified in this study fell within the Nigerian national estimates of 5% to 58.6% [3]. It is noteworthy that this study´s domestic abuse prevalence is much higher than that of developed nations [9], 9% in New Zealand [10] and 8% in Japan [11], but, similar to the reports from other developing nations such as India (48.2%) [31], and Ethiopia (78.5%) [14]. However, the study´s abuse rate is consistent with recent reports from other centres in Nigeria, such as Jos (31.8%) [21] and Jigawa (34.3%) [20]. But it is far higher than the 11% reported in the study area in 2003 [18]. This observed difference could be due to methodology differences, including the difference and accuracy of screening questionnaires. Also, the anonymity of our study´s process and the questionnaire's self-administered nature might have encouraged respondents' honesty and removed the possible fear of their responses being made public.

Furthermore, the higher prevalence of abuse might be related to the country's deteriorating socio-economic situation and the attendant increasing financial constraint. This explanation is supported by the respondents´ view that financial constraint was the most predominant cause of domestic abuse in the study area. Interestingly, the study also found that the unemployed status of the woman was an independent predictor of domestic abuse which might support the women´s view on the relationship between domestic abuse in pregnancy and financial constraints.

In the male-dominated society existing in the study area, it is surprising that the odds of experiencing domestic abuse by a parous woman was four times higher among women with at least a male child when compared to women without a son. Culturally, not having a son means a man will have no heirs and no one to carry on his name so, it was thought that a woman who is yet to bear a male child is at risk of abuse, not just from her spouse but also from her in-laws but, this study suggests otherwise. The explanation for this unexpected observation is not clear so further studies in this regard are recommended.

Most of the respondents were of the Igbo tribe, the predominant tribe in Nigeria's Southeast region. The Igbo culture seems to accept subordinate´s scolding; since men spend money (sometimes huge sums) and materials during marriages, it was not surprising that husbands were the foremost perpetrators of abuse in the study, while verbal abuse was the most common type of abuse in the index and previous pregnancies. Verbal abuse was the most common type of abuse in this study, which agreed with the previous study from the centre [18] but, differed from the findings from some other centres [4,15,17,20,23] where the physical battery was the most common form of abuse. These differences could be due to differences in cultural characteristics of the study populations. For instance, Northern Nigeria is a predominantly patriarchal society and has a culture of female seclusion. In some areas, beating a wife as a form of correction is the norm. In Igbo land, however, it is generally unacceptable to beat women, especially pregnant women. Nevertheless, compared to the previous study done in the study centre [18] where only 13.1% of respondents reported physical beating during pregnancy, this study found a rate of 32.1%. This difference may be explained by increased openness and awareness of abuse types by women, including beating. It used to be a social stigma if one admitted or reported being beaten by her husband, but more women and society, in general, are becoming aware of how wrong physical abuse is, and thus, more women are likely to be willing to speak up about it currently than in the past.

Forced sexual acts were not commonplace in this study, which contrasts a similar study in Jos [20], where spousal rape accounted for more than 65% of all abuse. This difference might be due to the varying cultural perspective about spousal rape. For instance, in the study area, it is culturally not believed that spousal rape exists; thus, this study´s spousal rape rate might have been grossly under-reported by respondents. The study also identified perceived complications of abuse experienced by the respondents during previous pregnancies - the commonest were preterm labour and depression. Interestingly, these findings were similar to the results of other related studies [4,15,17,20,23]. In the index pregnancies, the continuous feeling of sadness, which we described as depression, was the only complication reported by the respondents.

It is worrisome that most of the respondents were not aware of any existing laws that prohibited domestic abuse. Likewise, only 2% of respondents were aware of the Violence against Persons Prohibition (VAPP) act of 2015 [25], which is the most significant piece of legislation to date that addresses and allows for the prosecution of domestic abuse against women. Incidentally, respondents who were aware of this act during the study were legal practitioners, which casts doubt on the visibility of that legislation and calls for adequate public enlightenment by relevant government and non-government agencies. It is also noteworthy that a majority of respondents did not believe that the police should be trusted to handle domestic abuse matters. Thus, future programmes on domestic abuse should target improving case management by the Nigerian police force.

A limitation encountered in this study was the refusal by some women to participate because of the personal and sensitive nature of domestic abuse and the stigma attached to it. The use of a self-administered questionnaire that did not identify the respondent was provided to reduce this limitation. Also, more than half of the respondents had tertiary education which suggests that the women attending ANC at the university teaching hospital would probably be of certain socio-economic status when compared to other health care centres therefore, some level of selection bias might have occurred.

## Conclusion

Domestic abuse prevalence was high among pregnant women attending antenatal care at the University of Nigeria Teaching Hospital, Enugu, Nigeria. The most common perpetrator and types of abuse were women's spouses and verbal abuse, respectively. The predominant women perceived cause of abuse was financial constraints while the predictors among abused women were women's age of fewer than 25 years, unemployed status, educational status of less than tertiary education, and having at least a male child. Although most participants were not aware of their rights or the laws that protect victims of abuse or punish perpetrators, most of them recommended screening for domestic abuse during antenatal clinics.

Finally, domestic abuse is a threat to women´s reproductive health, and with the worsening socio-economic situation of Nigeria, the prevalence might be increasing considering the previous report from the study centre [18]. In line with respondents´ suggestion, we recommend that antenatal care centres, in our environment, should incorporate routine domestic violence screening and education on the existing domestic abuse laws into their services.

### What is known about this topic

- The prevalence and pattern of domestic abuse vary significantly, reflecting the genuine diversity in the definition of abuse in pregnancy by both the victims and the researchers;
- Financial constraints were the most common cause of domestic abuse in pregnancy in some homes in Enugu, Nigeria;
- The violence against persons (prohibition) act [VAPP act] bill is yet to be fully implemented at both the federal and state levels in Nigeria.

### What this study adds

- The overall domestic abuse prevalence was 43.0%, and the most common type of abuse being verbal abuse (85.5%), and 60% of the respondents believed that pregnant women should be screened for domestic abuse at the antenatal clinics;
- The predictors of domestic abuse identified by the study were - maternal age of fewer than 25 years, not having a tertiary education, having at least a male child, and the unemployed status of the respondent;
- Only 21.3% and 18.3% of respondents were aware that there were laws in Nigeria that prohibited domestic abuse and believed that abuse could be reported to the police respectively.
